# QTL Mapping of Grain Quality Traits Using Introgression Lines Carrying *Oryza rufipogon* Chromosome Segments in *Japonica* Rice

**DOI:** 10.1186/s12284-016-0135-0

**Published:** 2016-11-24

**Authors:** Yeo-Tae Yun, Chong-Tae Chung, Young-Ju Lee, Han-Jung Na, Jae-Chul Lee, Sun-Gye Lee, Kwang-Won Lee, Young-Hwan Yoon, Ju-Won Kang, Hyun-Sook Lee, Jong-Yeol Lee, Sang-Nag Ahn

**Affiliations:** 1 Chungcheongnamdo Agricultural Research and Extension Services, Yesan, 340-861 Korea; 2 College of Agriculture & Life Sciences, Chungnam National University, Daejeon, 305-764 Korea; 3 National Academy of Agricultural Sciences, Rural Development Admin., Jeonju, 560-500 Korea; 4 Department of Southern Area Crop Science, National Institute of Crop Science, Milyang, 50424 Korea

**Keywords:** Grain quality, Interspecific cross, Introgression lines, QTL, Rice

## Abstract

**Background:**

Improved eating quality is a major breeding target in *japonica* rice due to market demand. Consequently, quantitative trait loci (QTL) for glossiness of cooked rice and amylose content associated with eating quality have received much research focus because of their importance in rice quality.

**Results:**

In this study, QTL associated with 12 grain quality traits were identified using 96 introgression lines (IL) of rice developed from an interspecific cross between the Korean elite *O. sativa japonica* cultivar ‘Hwaseong’ and *O. rufipogon* over 7 years. QTL analyses indicated that QTL *qDTH6* for heading date, detected on chromosome 6 is associated with variance in grain traits. Most QTLs detected in this study clustered near the *qDTH6* locus on chromosome 6, suggesting the effect of *qDTH6. O. rufipogon* alleles negatively affected grain quality traits except for a few QTLs, including *qGCR9* for glossiness of cooked rice on chromosome 9. To characterize the effect of the *O. rufipogon* locus harboring *qGCR9,* four lines with a single but different *O. rufipogon* segment near *qGCR9* were compared to Hwaseong. Three lines (*O. rufipopgon* ILs) having *O. rufipogon* segment between RM242 and RM245 in common showed higher glossiness of cooked rice than Hwaseong and the other line (Hwaseong IL), indicating that *qGCR9* is located in the 3.4-Mb region between RM242 and RM245. Higher glossiness of cooked rice conferred by the *O. rufipogon* allele might be associated with protein content considering that three lines had lower protein content than Hwaseong (*P* < 0.1). These three *O. rufipogon* ILs showed higher yield than Hwaseong and Hwaseong IL due to increase in spikelets per panicle and grain weight indicating the linkage of *qGCR9* and yield component QTLs.

**Conclusion:**

The *qGCR9* locus is of particular interest because of its independence from other undesirable grain quality traits in *O. rufipogon*. SSR markers linked to *qGCR9* can be used to develop high-quality *japonica* lines and offer a starting point for map-based cloning of genes underlying this trait. To our knowledge, this is the first report to map a beneficial QTL for glossiness of cooked rice from a wild rice, *O. rufipogon*.

**Electronic supplementary material:**

The online version of this article (doi:10.1186/s12284-016-0135-0) contains supplementary material, which is available to authorized users.

## Background

Rice (*Oryza sativa*) is the most important staple food of more than half of the world’s population. The grain quality of this cereal crop has received major attention for its culinary and consumption value in recent years in several *japonica* growing countries including Korea and Japan (Kobayashi et al. 2008; Kwon et al. 2011). Rice cooking quality is an important factor driving market demand, as consumers prefer good, palatable rice (Kwon et al. 2011; Takeuchi et al. 2007; Wan et al. 2004). As the demand for high quality rice increases, the improvement of grain quality has become a major objective in rice breeding. However, selection for high quality rice is not easy due to complicated analyses and limited quantities of the grain in early generations of rice breeding programs. Thus, many studies have been conducted to understand the relationship between eating quality and grain traits. Several published reports have suggested that physicochemical characteristics, such as alkali digestion value, amylose content, and protein content, comprise an indirect method to estimate rice eating quality (Juliano 1971; Kobayashi et al. 2008; Wada et al. 2008). Eating and cooking quality of rice is mainly influenced by its starch, comprised of amylose and amylopectin. Amylose content is well recognized as the most important determinant of cooking and eating quality, and is controlled by the *waxy* gene on chromosome 6, which encodes the granule-bound starch synthase (Bao et al. 2000; He et al. 1999; Septiningsih et al. 2003; Tan et al. 1999).

Eating quality is characterized by cohesiveness, hardness, and other features that are largely controlled by amylose content, gel consistency, gelatinization temperature, and protein content (Bao et al. 2004; Juliano 1971). Protein is the second most abundant storage matter in rice endosperm, averaging 8% as dry seed weight (Gomez 1979). It is accepted that high protein levels generally have a deleterious effect on physico-chemical properties of cooked rice and protein content is negatively correlated with eating quality (Juliano et al. 1965; Kwon et al. 2011; Wada et al. 2006) and positively correlated with hardness of cooked rice (Okadome 2005).

Most grain quality traits are quantitatively inherited, and it is difficult for breeders to select for quality using conventional methods mainly due to the time-consuming and expensive assay methods. Assessment of grain quality is further complicated by epistasis, the triploid nature of endosperm, and environmental effects (He et al. 1999). The use of molecular markers has facilitated our understanding of the genetic mechanisms undergirding complex quantitative traits in crops. Numerous QTL analyses for rice quality traits have been conducted to identify and characterize QTLs using various mapping strategies and populations (Aluko et al. 2004; Bao et al. 2000, 2004; Kwon et al. 2011; Sabouri et al. 2012; Takeuchi et al. 2007, 2008; Zhang et al. 2013). Several studies have revealed a link between heading date (flowering time) and grain quality (Kwon et al. 2011; Hori et al. 2012). Indeed, heading date determines the mean temperature during the grain filling stage, which in turn impacts rice quality. To identify stable QTLs which are not altered easily by environmental factors, materials for QTL analysis should be similar or identical in heading date to eliminate confounding effects of the environment.

Breeding efforts to utilize wild species of rice are active as a way to increase yield, crop stability, and to broaden the gene pool (Brar and Khush 1997; Xie et al. 2008). Numerous genes conferring resistance to diseases and insect pests have been identified in wild species (Brar and Khush 1997; Suh et al. 2014). ‘Anmi’ is a new BPH (Brown planthopper) resistant *japonica* rice cultivar possessing the *Bph18* gene derived from wild rice, *Oryza australiensis* and high yield potential with good grain quality (Suh et al. 2014). These results suggest that rice productivity and stability would be improved by transferring the useful gene from wild rice into the cultivar. However, there is little effort to improve rice grain quality traits using wild species of rice (Aluko et al. 2004; Brondani et al. 2002; Yuan et al. 2010).

The objectives of this study were 1) to identify QTLs associated with rice quality traits using 96 introgression lines (ILs) derived from a cross between *O. sativa* Hwaseong and *O. rufipogon*, 2) to determine the effect of heading date on grain quality traits, 3) to understand the genetic basis of grain quality traits, and 4) to validate the effects of QTLs for glossiness of cooked rice on chromosome 9.

## Results

### Genetic Characterization of Introgression Lines (ILs)

**Fig. 1:**
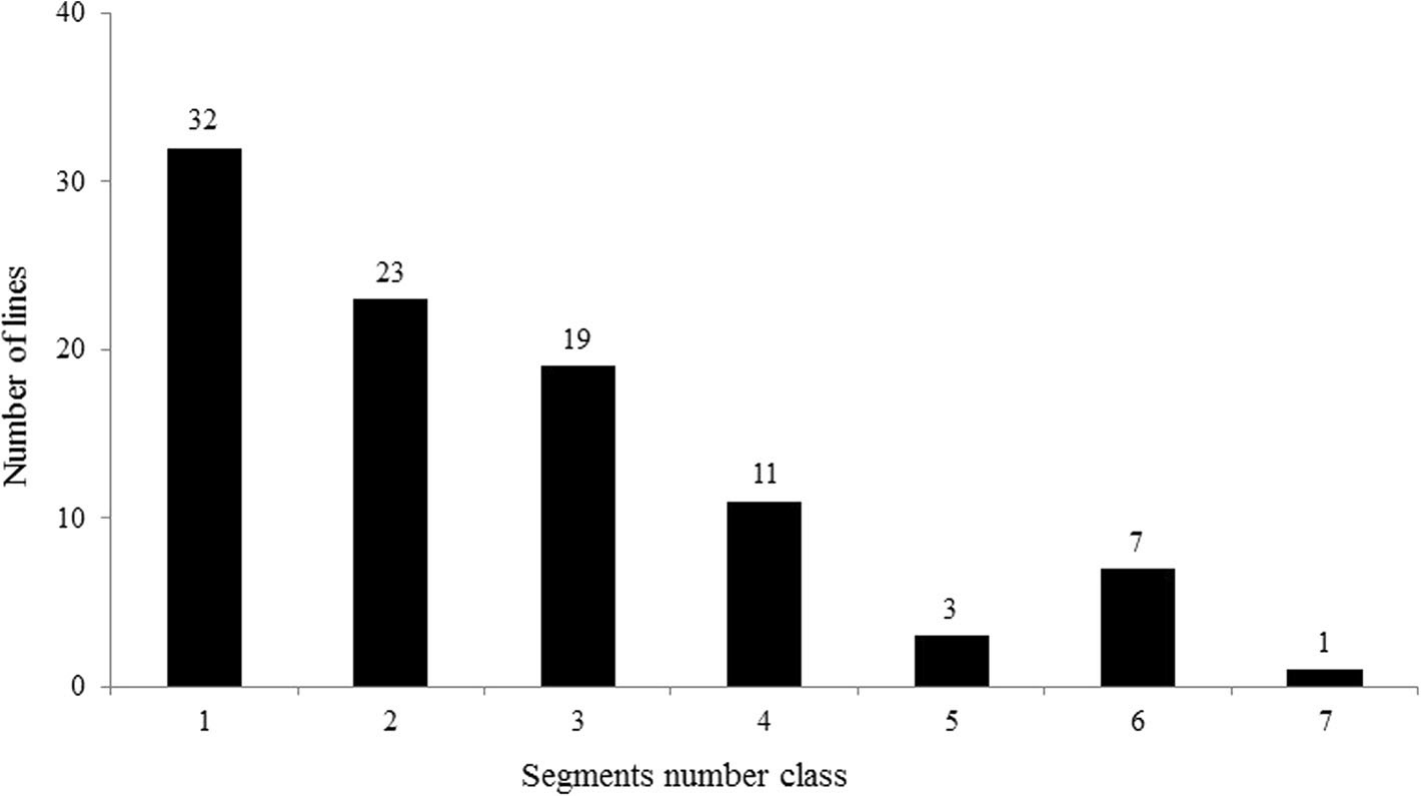
Frequency distribution of the introgression segments per each IL

A total of 241 introgressions, including 207 homozygous and 34 heterozygous segments, were detected on 12 chromosomes based on the genotypes of 133 SSR markers in 96 ILs (Additional file 1: Table S1). Each of the 96 ILs contained 0–7 homozygous and 0–3 heterozygous introgressions (Fig. 1). The ratio of homozygous to heterozygous introgression segments varied among the 12 rice chromosomes. Chromosome 8 was found to have the highest number of introgressions, while chromosome 5 revealed no introgression events. Ninety six ILs represented 66% of the *O. rufipogon* genome. The recurrent parent genome in ILs ranged from 86 to 99%, with a mean of 96%.

### Phenotypic Characterization of ILs

Traits For all ILs, grain quality traits, including HR (head rice ratio), CR (chalky rice ratio), PC (protein content), AC (amylose content), and GCR (glossiness of cooked rice), recorded for 7 years, and starch viscosity characteristics measured for 5 years (2009 to 2013) are summarized in Table 1. The starch viscosity characteristics are pasting temperature (PST), peak viscosity (PKV), hot paste viscosity (HPV), cool paste viscosity (CPV), breakdown viscosity (BDV = PKV − HPV), setback viscosity (SBV = CPV − PKV), and consistency viscosity (CSV = CPV − HPV).

**Table 1:** Comparison of 13 traits between two parents and among three groups

Traits^a)^		Mean			Mean ± S.D			ANOVA^f)^		
		Hwaseong	*O. rufipogon*	Diff.^b)^	96 lines^c)^	83 lines^d)^	13 lines^e)^	Year (Y)	Genotype (G)	Y × G
Days to heading		108	109	ns	105 ± 6b ^g)^	107 ± 3b	94 ± 4a			
Rice quality	HR (%)	85	26	**	82.7 ± 13.4b	85.7 ± 10.2b	63.8 ± 15.9a	22.1**	325.2**	3.3**
	CR (%)	6.2	34.7	**	10.0 ± 11.7a	7.9 ± 8.7a	23.4 ± 17.8b	31.1**	664.2**	4.8**
	PC (%)	6.0	7.2	**	6.3 ± 0.6a	6.2 ± 0.5a	7.1 ± 0.6b	5.9**	32.1**	4.1**
	AC (%)	18.8	25.3	**	18.5 ± 0.7b	18.6 ± 0.5b	17.5 ± 0.7a	6.3**	229.1**	1.9**
	G C R	69.2	44.8	**	68 ± 7b	69 ± 5b	59 ± 10a	7.5**	152.1**	1.6**
Starch viscosity	PST (°C)	75.4	79.3	**	77.4 ± 5.4a	76.9 ± 5.5a	80.2 ± 3.6b	1.7**	186.2**	1.1^ns^
	PKV (RVU)	244.5	177.9	**	234.4 ± 34.9a	230.9 ± 29.3a	255.9 ± 55.3b	6.9**	765.1**	1.1 ^ns^
	HPV (RVU)	133.9	131.1	ns	138.5 ± 19.6	137.5 ± 18.4	145.0 ± 25.4	10.0**	755.2**	1.0 ^ns^
	BDV (RVU)	88.6	57.8	**	95.7 ± 22.8a	93.4 ± 19.5a	110.9 ± 34.8b	3.9**	1013.0**	0.7 ^ns^
	CPV (RVU)	236.3	185.8	**	243.7 ± 30.1a	242.7 ± 28.9a	250.6 ± 36.5a	5.2**	11.0**	0.6 ^ns^
	SBV (RVU)	−18.22	7.9	**	9.5 ± 22.1b	11.7 ± 17.9b	−5.4 ± 36.9a	4.1**	97.1**	0.6 ^ns^
	CSV (RVU)	102.4	54.7	**	105.0 ± 14.5a	105.0 ± 13.8a	104.6 ± 18.4a	2.8**	1208.0**	0.6 ^ns^

a) *HR* Head rice ratio, *CR* Chalky rice ratio, *PC* Protein content, *AC* Amylose content, *GCR* Glossiness of cooked rice, *PST* pasting temperature, *PKV* peak viscosity, *HPV* hot paste viscosity, *BDV* breakdown viscosity, *CPV* cool paste viscosity, *SBV* setback viscosity, *CSV* consistency viscosity. b) Difference between Hwaseong and *O. rufipogon*. ns, *, and **indicate not significant, significant at *P* < 0.05 and *P* < 0.01, respectively. c) all the lines, d) 86 introgression possessing no *O. rufipogon* segments on the region of *qDTH6*, e) 13 introgression lines possessing the *O. rufipogon* segment on the region of *qDTH6.* f) ANOVA with 96 lines over 7 years. ns, *, and **indicate not significant, significant at *P* < 0.05 and *P* < 0.01, respectively. g) Numbers followed by the same letter in each row are not significantly different based on Duncan’s multiple range test at *P* < 0.05

#### Rice Grain Quality Traits

**Fig. 2:**
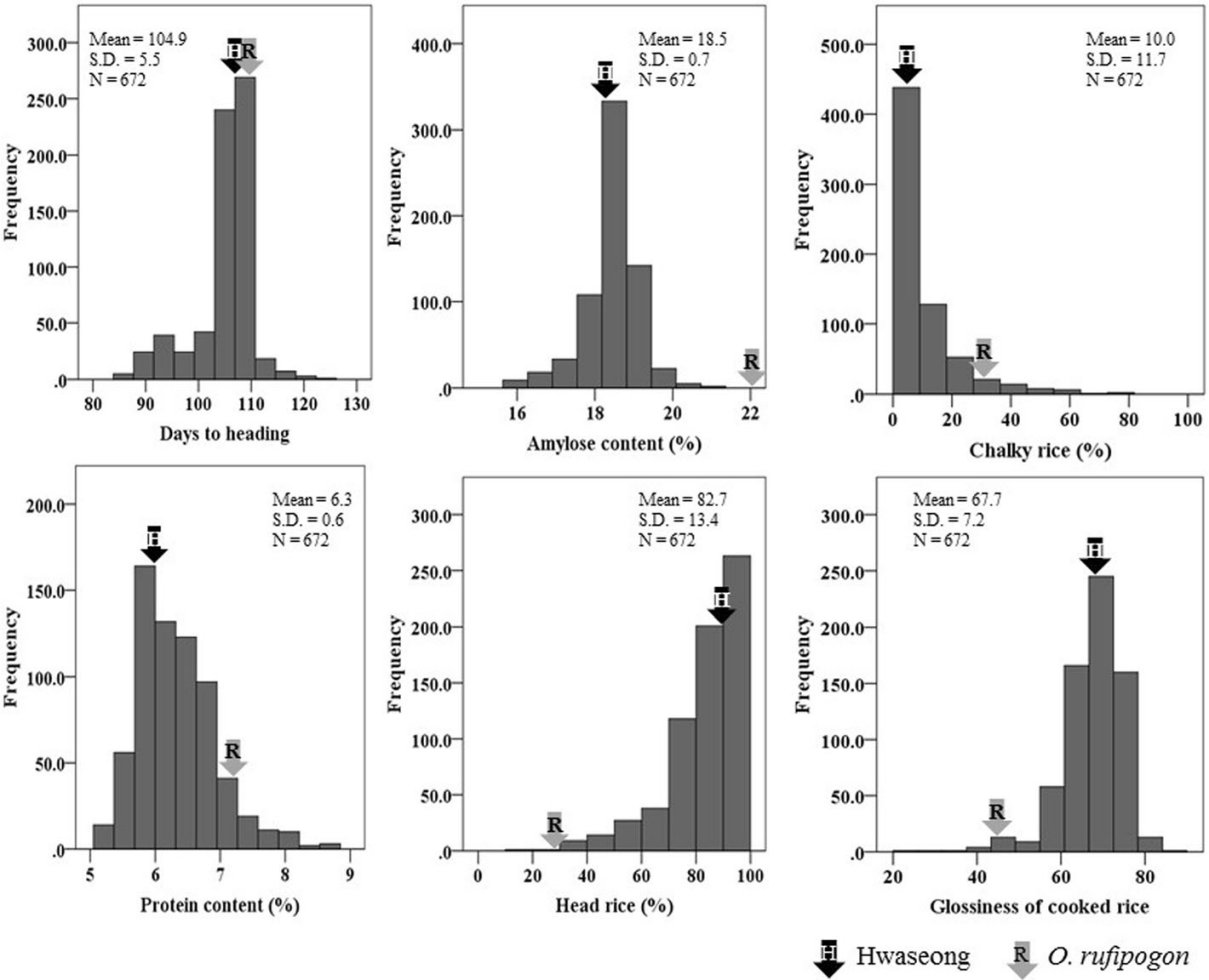
Frequency distribution of 96 ILs with two parents for days to heading and five grain quality traits over 7 years. Hwaseong  *O. rufipogon*

Differences in grain quality traits between the parents were highly significant for all traits (Table 1). Compared to *O. rufipogon*, Hwaseong had higher head rice ratios and glossiness of cooked rice, but lower chalky rice ratios, amylose content, and protein content. For 96 ILs phenotyped over 7 years, head rice and chalky rice ratio metrics showed a continuous distribution with a negative and positive skew, respectively, while protein content, amylose content, and glossiness of cooked rice followed a normal distribution (Fig. 2). Transgressive segregation that fell beyond the high or low mean of the two parents was observed, and the mean trait values of the ILs were nearly the same as those of Hwaseong. An ANOVA for the five rice quality traits revealed significant variation by genotype (G) and year (Y), and the interaction of G × Y was also highly significant (Table 1).

#### Starch Viscosity Characteristics

**Fig. 3:**
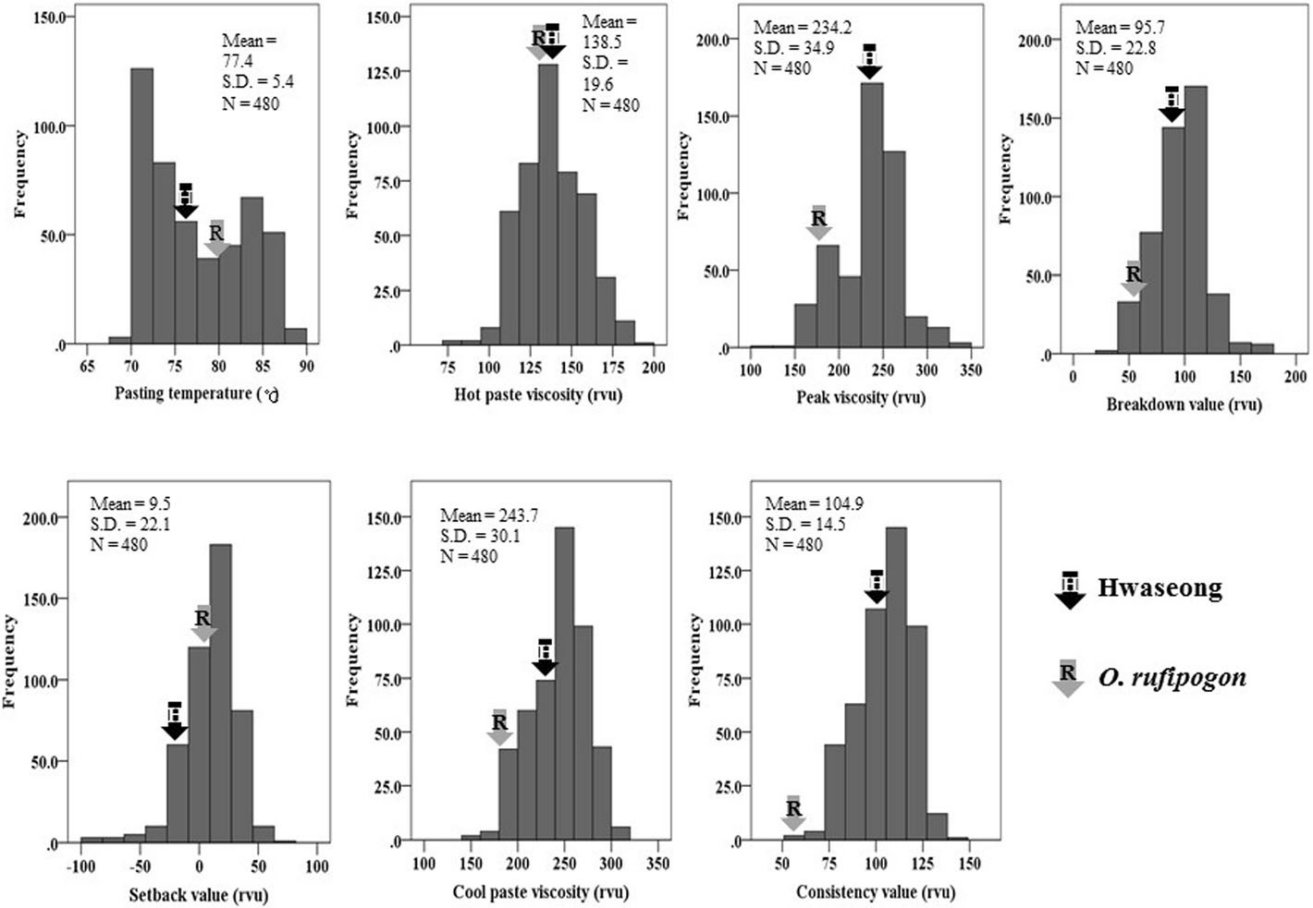
Frequency distribution of 96 ILs with two parents for seven starch viscosity characters over 5 years. Hwaseong  *O. rufipogon*

Starch viscosity characteristics of the parents and 96 ILs obtained from the RVA analyzer are shown in Table 1. A large difference in most of the starch viscosity characteristics was observed between the parents, except for hot paste viscosity. Compared to *O. rufipogon*, Hwaseong showed higher peak viscosity, breakdown viscosity, cool paste viscosity, and consistency of starch, whereas Hwaseong was found to be lower in pasting temperature and setback viscosity. The frequency distribution of starch viscosity characteristics for the 96 ILs is shown in Fig. 3. Traits such as HPV, CPV, and SBV showed normal distributions. For all trait values, transgression lines that fell beyond the high or low mean of the two parents were observed. The mean trait values of the ILs were nearly the same as Hwaseong. An ANOVA (analysis of variance) for starch viscosity characteristics revealed significant variation by genotype and year, respectively (Table 1). However, no significant G × Y interaction was observed.

### Correlation Analysis

Correlation values among rice quality and starch viscosity characteristics are summarized in Table 2. Significant correlations between rice quality and starch viscosity characteristics were observed. Head rice ratio was found to be positively correlated with amylose content and glossiness of cooked rice, but negatively correlated with chalky rice ratio and protein content. AC was positively correlated with SBV and CSV, and negatively correlated with PST, PKV, and BDV. GCR was positively correlated with CPV, SBV, and CSV and negatively correlated with PST. The highest positive and negative correlations among rice quality traits was observed between HR and GCR (*r* = 0.51**), and HR and CR (*r* = −0.86*), respectively. Breakdown viscosity was positively correlated with PKV, HPV, CPV and CSV, but negatively correlated with PST and SBV. The highest positive and negative correlations in starch viscosity characteristics was observed between CPV and HPV (*r* = 0.92*) and BDV and SBV (*r* = −0.80**), respectively. These results suggest that starch viscosity characteristics are dependent on one another. Also, significant correlations between rice quality and starch viscosity characteristics were observed. AC was positively correlated with SBV and CSV, and negatively correlated with PST, PKV, and BDV. GCR was positively correlated with PT, CPV, SBV, and CSV.

**Table 2:** Correlation coefficient among rice quality traits and starch viscosity characteristics

Trait^x)^	DTH	HR	CR	AC	PC	GCR	PST	PKV	HPV	BDV	CPV	SBV
HR	0.587** ^y)^											
CR	−0.488**	−0.860**										
AC	0.539**	0.424**	−0.359**									
PC	−0.536**	−0.416**	0.360**	−0.368**								
GCR	0.484**	0.510**	−0.497**	0.455**	−0.621**							
PST	−0.212**	−0.393**	0.301**	−0.115*	0.242**	−0.139**						
PKV	−0.213**	0.102*	−0.211**	−0.159**	0.071 ^ns^	0.008 ^ns^	−0.271**					
HPV	−0.192**	0.078^ns^	−0.215**	−0.061 ^ns^	0.071 ^ns^	0.089 ^ns^	0.001 ^ns^	0.790**				
BDV	−0.150**	0.107*	−0.141**	−0.190**	0.042 ^ns^	−0.06 ^ns^	−0.414**	0.850**	0.349**			
CPV	−0.123**	0.174**	−0.335**	0.007 ^ns^	−0.013 ^ns^	0.117*	−0.055 ^ns^	0.778**	0.923**	0.397**		
SBV	0.158**	0.057^ns^	−0.121**	0.259**	−0.124**	0.141**	0.352**	−0.519**	0.001 ^ns^	−0.802**	0.134**	
CSV	0.020 ^ns^	0.276**	−0.412**	0.100*	−0.133**	0.122**	−0.137**	0.540**	0.558**	0.347**	0.818**	0.261**

^x)^Refer to Table 1 for trait abbreviations. ^y)^ *, **: Significant at *P* < 0.05 and *P* < 0.01, respectively. ^ns^: not significant

### Sequence Comparison of *Wx* and *ALK* Genes Between Two Parents

The allelic differences of *Wx* and *ALK* genes between ‘Hwaseong’ and *O. rufipogon* were investigated to analyze their possible effects on the properties of starch and quality. Alignment of genomic sequences of the *Wx* gene between Hwaseong and *O. rufipogon* generated by the PCR primer set, WP-A2 and WP-B (Yamanaka et al. 2004) showed that Hwaseong possessed *Wx* ^b^ allele with a G to T mutation of intron 1 at the *Wx* gene whereas *O. rufipogon* had *Wx* ^a^ allele. For the *ALK* gene, two SNPs in the coding region showing association with alkali spreading value and gelatinization temperature were assayed by using two PCR primer sets, ALKSNP3-AF and ALKSNP4-GCR for SNP3 and ALKSNP3-GF and ALKSNP4-TTR for SNP4 (Gao et al. 2011). Sequence alignment showed that Hwaseong possessed the same sequences as TN1 at SNP3 (G) and SNP-4 (TT) position and *O. rufipogon* shared the same sequences as Minghui 63 at SNP3 (G) and SNP-4 (GC) position.

### QTL Identification

To determine whether days to heading (DTH) affects variance in rice quality traits, QTL analysis was conducted to detect QTL for heading date. A major heading date QTL, *qDTH6*, was detected on chromosome 6. Also, phenotypic data showed that DTH was highly correlated with mean temperature during the grain filling stage, which influenced both agronomic and rice quality traits (data not shown). To understand the effect of heading date on rice grain quality, we compared the variance between the two groups (all 96 ILs and 83 ILs lacking the *qDTH6* QTL). Mean values of rice quality traits, including starch viscosity characteristics, for each group are summarized in Table 1. Differences among the groups were detected in rice quality traits. Thirteen lines with *qDTH6* flowered earlier than lines without in both groups, by 11 and 13 days, respectively. Thirteen lines showed higher values in chalky rice ratio and protein content, and lower values of glossiness of cooked rice and head rice ratio than the 96 and 83 lines, mainly due to the effect of the *O. rufipogon qDTH6* allele contributing to early heading.

**Fig. 4:**
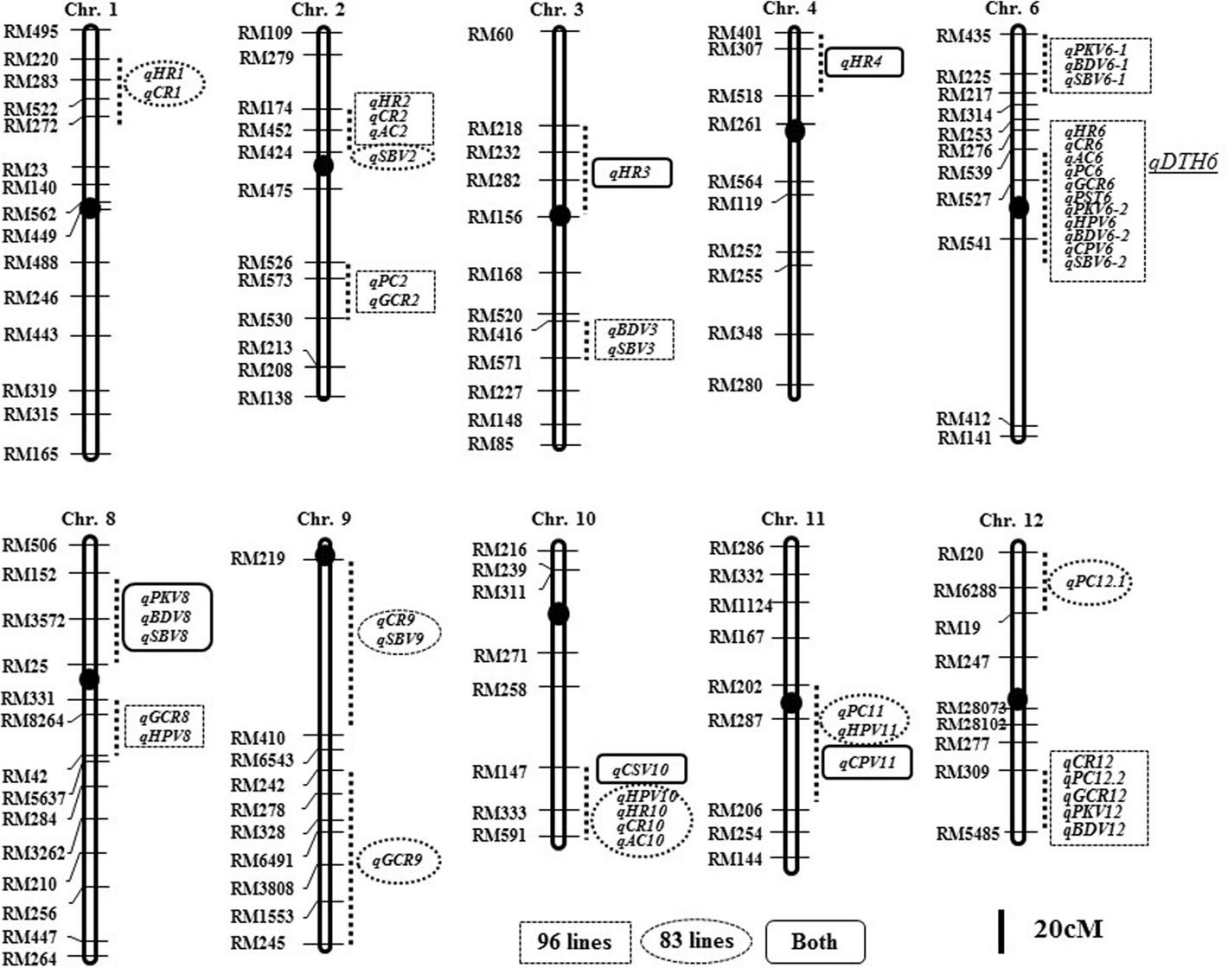
Map locations of the QTLs detected in this study. QTLs detected by single point analysis and interval mapping were represented to the right of the chromosome. QTLs detected in both groups, 96 ILs and 83 ILs are shown. *qDTH6* for days to heading is shown on chromosome 6

A total of 25 QTLs for rice quality and 23 for starch viscosity characteristics were detected for these 12 traits and summarized (Tables 3 and 4, Fig. 4). Of the 48 QTLs identified in total, 35 QTLs were detected in 96 lines, 20 QTLs in 83 lines, and 7 QTLs were commonly identified in two groups (96 and 83 ILs). Most of the QTLs detected in this study were located in the same or adjacent regions as those reported by previous studies, and clustered in several regions. The *O. rufipogon* alleles showed negative effects on the traits at most of the QTLs (Tables 3 and 4).

**Table 3:**
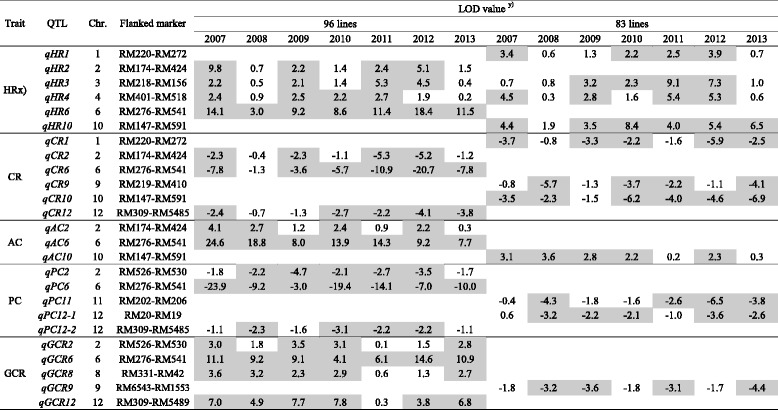
QTL information for rice quality traits in 96 and 83 introgression lines

^x)^Refer to Table 1 for trait abbreviations. ^y)^QTL peak with LOD ≥ 2.1 are colored with gray, and negative values indicate that the *O. rufipogo*n alleles increased the trait values

**Table 4:**
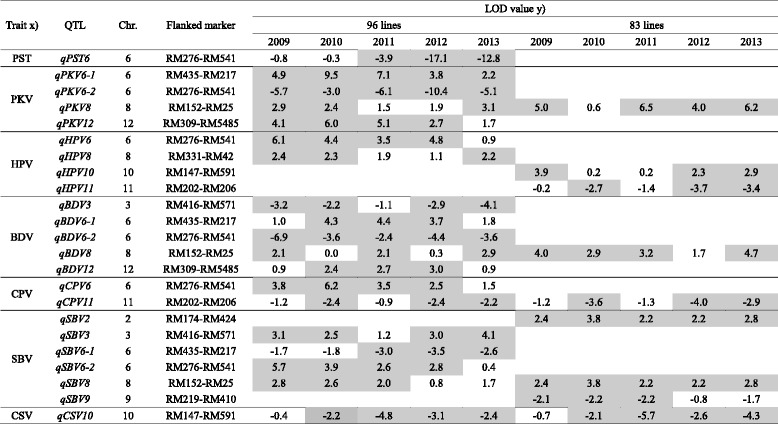
QTL information for starch viscosity characteristics in 96 and 83 introgression lines

^x)^Refer to Table 1 for trait abbreviations. ^y)^QTL peak with LOD ≥ 2.1 are colored with gray, and negative values indicate that the *O. rufipogo*n allele increased the trait values

#### QTL for Agronomic Traits

##### DTH (Days to Heading)

A QTL *qDTH6,* associated with DTH, was identified near markers RM539 and RM527 on chromosome 6 for seven consecutive years, explaining from 42 to 78% of the total phenotypic variance (data not shown). This QTL was detected in a similar genomic location by Yano et al. (2000). The *O. rufipogon* allele at *qDTH6* decreased the number of DTH.

#### QTLs for Rice Quality Traits

A total of 25 QTLs for rice quality traits were identified in this study. Of these, only two QTLs, *qHR3 and qHR4*, were commonly identified in both groups (96 and 83 ILs). Moreover, 16 QTLs were detected in 96 ILs with 11 QTLs in 83 ILs (Table 3).

##### HR (Head Rice Ratio)

Six QTLs for head rice ratio were identified in both groups. Of these, *qHR3-2* and *qHR4* were commonly detected in both groups and explained 9 to 42% of the phenotypic variance. At all six loci, *O. rufipogon* alleles decreased the head rice ratio.

##### CR (Chalky Rice Ratio)

Six QTLs for chalky rice ratio were identified in both groups. Of these, three QTLs, *qCR2, qCR6,* and *qCR12* were detected in 96 ILs, and three other QTLs, *qCR1*, *qCR9,* and *qCR10,* were detected in 83 ILs. At all six loci, *O. rufipogon* alleles increased the chalky rice ratio.

##### AC (Amylose Content)

Three QTLs for amylose content were identified in this study. Two QTLs, *qAC2 and qAC6,* were identified in 96 ILs, and one QTL, *qAC10,* was detected in 83 ILs. At three loci, *O. rufipogon* alleles decreased amylose content. The QTL *qAC10* was found to explain 12 to 18% of the phenotypic variance.

##### PC (Protein Content)

Five QTLs for protein content were identified in this study. Of the five QTLs detected, *qPC11* and *qPC12-1* were detected in 83 ILs, while the other three QTLs, *qPC2*, *qPC6,* and *qPC12-2* were found in 96 ILs. At all loci, *O. rufipogon* alleles increased the protein content.

##### GCR (Glossiness of Cooked Rice)

Of the five QTLs for glossiness of cooked rice, four QTLs, *qGCR2*, *qGCR6*, *qGCR8*, and *qGCR12* were identified in 96 ILs, and at these loci, *O. rufipogon* alleles decreased the glossiness of cooked rice value. One QTL, *qGCR9* detected on chromosome 9 in 83 lines, was not reported in previous studies. At this locus, the *O. rufipogon* allele increased the glossiness of cooked rice.

#### Starch Viscosity Characters

A total of 23 QTLs for starch viscosity traits were identified in this study. Of these, five QTLs, *qPKV8, qBDV8, qCPV11, qSBV8,* and *qCSV10* were commonly identified in both groups. In addition, 19 QTLs were detected in 96 ILs and 9 QTLs were in 83 ILs (Table 4).

##### PST (Pasting Temperature)

One QTL for pasting temperature was identified in the 96 ILs. At *qPST6,* the *O. rufipogon* allele increased the pasting temperature and explained 18.2 to 57.1% of the phenotypic variance.

##### PKV (Peak Viscosity)

Four QTLs were identified in both groups. QTL *qPKV8* was commonly detected in both groups, and the three other QTLs, *qPKV6-1, qPKV6-2,* and *qPKV12,* were detected in 96 ILs. The *O. rufipogon* allele at *qPKV6-2* increased the peak viscosity, but *O. rufipogon* alleles at *qPKV6-1, qPKV8,* and *qPKV12* decreased the value. *qPKV8* shared a similar genomic location to the QTL detected by Bao et al. (2000).

##### HPV (Hot Paste Viscosity)

Four QTLs for hot paste viscosity were identified in both groups. Two QTLs, *qHPV6* and *qHPV8,* were observed in 96 ILs, and *qHPV10* and *qHPV11* were detected in 83 ILs. *O. rufipogon* alleles decreased the hot paste viscosity at *qHPV6*, *qHPV8,* and *qHPV10,* but increased the hot paste viscosity at *qHPV11*.

##### BDV (Breakdown Viscosity)

Five QTLs for breakdown viscosity were identified in both groups. QTL *qBDV8* was detected commonly in both groups, and four QTLs, *qBDV3, qBDV6-1, qBDV6-2,* and *qBDV12*, were detected in 96 ILs. At two QTLs, *qBDV3* and *qBDV6-2,* the *O. rufipogon* alleles increased the breakdown viscosity, while at the other three loci, *qBDV6-1, qBDV8,* and *qBDV12,* it decreased the breakdown viscosity.

##### CPV (Cool Paste Viscosity)

Two QTLs for cool paste viscosity were identified in both groups. One QTL, *qCPV6*, was detected in 96 ILs, and *qCPV1* was commonly detected in both groups. At all loci, the *O. rufipogon* alleles increased the cool paste viscosity.

##### SBV (Setback Viscosity)

Six QTLs for setback viscosity were identified in both groups. QTL *qSBV8* was commonly detected in both groups, and three QTLs, *qSBV3, qSBV6-1,* and *qSBV6-2*, were observed in 96 ILs, with *qSBV2* and *qSBV9* in 83 ILs. At *qSBV6-1* and *qSBV-9*, the *O. rufipogon* alleles increased the setback viscosity, but decreased it at the other loci.

##### CSV (Consistency Viscosity)

A previously unidentified QTL for consistency viscosity was commonly identified in both groups. At *qCSV10*, *O. rufipogon* allele increased the consistency viscosity.

### Characterization of *qGCR9*

**Fig. 5:**
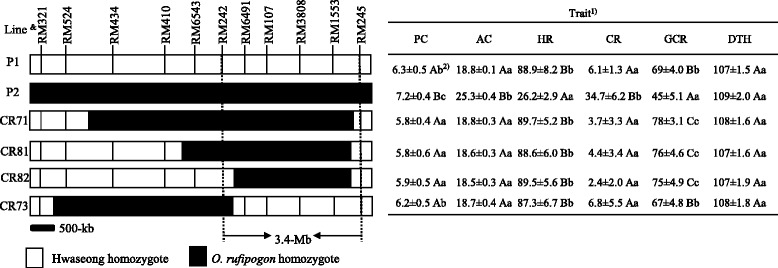
Graphical representation of four ILs and a map of the target region for glossiness of cooked rice QTL, *qGCR9* on chromosome 9. *White* and *black* portions of the graph are homozygous Hwaseong and homozygous *O. rufipogon*, respectively. The table to the *right* of the graphical genotypes indicates mean values of traits. Years were treated as replications. & P1: Hwaseong, P2: *O. rufipogon*. ^1)^ *PC* protein content, *AC* amylose content, *HR* head rice ratio, *CR* chalky rice ratio, *GCR* glossiness of cooked rice, *DTH* days to heading. ^2)^ *Numbers* followed by the same *letter* in each column are not significantly different based on Duncan’s multiple range test. A, B: ranked by Duncan test at *P* < 0.05. a, b: ranked by Duncan test at *P* < 0.1

*qGCR9* improved the glossiness of cooked rice in the Hwaseong cultivar background (Table 3). To characterize and further define the location of *qGCR9*, four lines with a single *O. rufipogon* segment near *qGCR9* on chromosome 9 were selected and screened with additional SSR markers (Fig. 5). Of the four ILs, three ILs, CR71, CR81, and CR82 with significantly higher values of glossiness of cooked rice than the control Hwaseong, shared the *O. rufipogon* segment between RM242 and RM245 in common, whereas CR73 with the *O. rufipogon* segment between RM321 and RM6491 showed no difference in the glossiness of cooked rice with Hwaseong. These results suggest that the 3.4-Mb *O. rufipogon* segment between RM242 and RM245 is associated with an increase in glossiness of cooked rice. Although CR20 and CR56 possssed an *O. rufipogon* segment near *qGCR9*, they were not included in the analysis because they had *O. rufipogon* segments in Hwaseong background. No significance difference in the glossiness of cooked rice between CR20 (66.1) and Hwaseong (66.1 and 69.0), and CR56 and Hwaseong (68.9 and 69.0) was observed and these two lines will be useful to further narrow down the candidate region. The protein content values of these three lines were lower than Hwaseong (*P* < 0.1) indicating that protein content might be associated with the variation of GCR; however, no difference in amylose content, head rice ratio, days to heading or chalky rice ratio was observed. Four ILs, with different *O. rufipogon* DNA segments in the target region were used for yield trials together with Hwaseong over 7 years. The yield trials were conducted using a completely randomized block design with two replications (Table 5). Three lines, CR71, CR81, and CR82 performed better than Hwaseong in spikelets per panicle and grain yield. However, no difference was observed for grain shape traits of rough and brown rice, grain length, width, thickness, and weight. Results showed that the average grain yield per plant of three lines CR71, CR81, and CR82 were 3.4–10.6% and 5.6–13.0% higher than that of CR73 and Hwaseong, respectively. The difference in grain yield per plant was significant (*P* ≤ 0.05) between three ILs (CR71, CR81, and CR82) and CR73 and Hwaseong, but there was no significant difference between CR73 and Hwaseong (Fig. 5). Same results were obtained for spikelets per panicle. No difference was observed in ripening ratio and panicle number (data not shown). These results indicate that the yield increase in three lines, CR71, CR81, and CR82 is mainly due to an increase in spikelets per panicle.

**Table 5:** Comparison of the grain shape and yield traits among two parents and four ILs

Line	Rough rice				Brown rice				SPP^c^ (no.)	GY^d^ (g/plant)
	Length (mm)	Width (mm)	Thickness (mm)	TGW^b^ (g)	Length (mm)	Width (mm)	Thickness (mm)	TGW (g)		
Hwaseong	7.3 ± 0.2 a^a^	3.2 ± 0.1 b	2.3 ± 0.05 b	26.2 ± 0.8 a	5.3 ± 0.2 a	2.8 ± 0.1 b	2.0 ± 0.05 b	22.1 ± 0.6 a	102 ± 6.5 b	23.6 ± 0.11 b
*O. rufipogon*	8.4 ± 0.2 b	2.9 ± 0.1 a	2.1 ± 0.04 a	27.2 ± 0.9 b	6.0 ± 0.2 b	2.6 ± 0.1 a	1.9 ± 0.05 a	21.9 ± 0.3 a	89 ± 7.1 a	21.3 ± 0.44 a
CR71	7.3 ± 0.3 a	3.3 ± 0.1 b	2.2 ± 0.09 b	26.5 ± 0.6 a	5.2 ± 0.2 a	2.9 ± 0.1 b	2.1 ± 0.09 b	22.3 ± 0.6 a	121 ± 9.0 c	24.4 ± 0.56 c
CR81	7.4 ± 0.3 a	3.2 ± 0.1 b	2.2 ± 0.07 b	26.3 ± 0.6 a	5.3 ± 0.1 a	2.9 ± 0.1 b	2.0 ± 0.06 b	22.3 ± 0.6 a	123 ± 8.9 c	26.1 ± 0.57 c
CR82	7.3 ± 0.2 a	3.2 ± 0.1 b	2.2 ± 0.09 b	26.5 ± 0.7 a	5.1 ± 0.1 a	2.8 ± 0.1 b	2.0 ± 0.08 b	22.4 ± 0.8 a	131 ± 9.1 c	25.3 ± 0.54 c
CR73	7.1 ± 0.2 a	3.1 ± 0.1 b	2.2 ± 0.07 b	25.9 ± 0.5 a	5.1 ± 0.1 a	2.8 ± 0.1 b	1.9 ± 0.07 b	21.8 ± 0.4 a	108 ± 9.6 b	23.1 ± 0.67 b

^a^Numbers followed by the same letter in each column are not significantly different based on Duncan’s multiple range test. ^b^1000 grain weight, ^c^spikelets per panicle, ^d^Grain yield in brown rice

## Discussion

Rice quality is an important consideration in the face of increased demand for high quality *japonica* rice (Kobayashi et al. 2008; Kwon et al. 2011). Therefore, breeding high quality rice is the top priority in breeding programs, and many studies with the aim to understand the genetic behavior of grain quality traits and their relationship to other traits have been conducted over the last few decades (Kobayashi et al. 2008; Kwon et al. 2011; Takeuchi et al. 2007).

It is generally accepted that rice grain traits are affected by temperature during grain filling, with high temperatures decreasing head rice ratio and increasing chalky rice ratio (Kobayashi et al. 2008; Kwon et al. 2011; Nishimura et al. 1985). A number of studies have been conducted to detect QTLs for rice quality traits (Aluko et al. 2004; Bao et al. 2000, 2004; Kwon et al. 2011; Sabouri et al. 2012; Yuan et al. 2010) and studies reported the co-localization of QTLs for heading date and grain quality traits (Wada et al. 2006; Hori et al. 2012). Heading date affected the chemical components, including alkali digestion value and amylose and protein content, which could affect eating quality (Hori et al. 2012; Nishimura et al. 1985; Wada et al. 2006). To investigate the effect of heading date on rice grain quality traits, we compared the variation of traits between two groups, 96 ILs as one group and the other group consisting of 83 ILs lacking *qDTH6*, a major heading date QTL. The number of days to flowering in 96 lines was 2 days earlier than that in 83 lines. Both groups showed differences in mean values for head rice ratio and chalky rice ratio, whereas no difference was observed for the other traits. The QTLs for grain traits also showed differences in position and number between two groups. Of the 48 QTLs, 35 QTLs were detected in 96 lines (with 14 QTLs located near *qDTH6*), whereas 20 QTLs were identified in 83 lines. Only 7 QTLs (14.6%) were commonly identified in both groups, and these results indicate that heading date affects rice grain traits. At the *ALK* locus 13 early heading lines possessed the *O. rufipogon* allele with the same SNPs as Minghui 63 with lower alkali spreading value than Hwaseong. It has been observed that the *ALK* affected paste viscosity parameters and gelatinization temperature (Gao et al. 2011; Wang et al. 2007). These findings raise the possibility that the *ALK* is responsible for the colocalization of paste viscosity parameters in this region which should be tested by using near-isogenic lines.

It is noteworthy that the *O. rufipogon* alleles at all QTL decreased AC although *O. rufipogon* showed higher AC than Hwaseong. Sequence comparison indicated that Hwaseong possessed *Wx* ^b^ allele with a G to T mutation of intron 1 at the *Wx* gene whereas *O. rufipogon* had *Wx* ^a^ allele. There are at least two possible explanations for these observations. One is that the *O. rufipogon* segments associated with an increase in AC were not introgressed into the 96 lines and additional genetic factors for AC were not detected. For example, no *O. rufipogon* segment was detected on the entire chromosome 5 and some portion of the long arm of chromosome 3 due to negative selection, and other handling problems during generation advancement. Several studies reported QTLs for AC in these chromosomal regions (He et al. 1999; Tanaka et al. 2006; Edabi et al. 2013). Another is that AC was greatly influenced by the alteration of heading date and the QTL cluster on chromosome 6 represents the pleiotropic effect of the heading date QTL. A number of studies reported positive correlations between AC and the number of days to heading (Tanaka et al. 2006; Wada et al. 2006). Early heading lines would have short period of vegetative growth and experience higher mean temperature during ripening than late heading line in Korea. It has been previously reported that amylose content in *japonica* rice cultivars often decreased when grain developed at relatively high temperatures than at low temperatures whereas *indica* varieties containing the *Wx* ^*a*^ allele are not sensitive temperature with no distinct correlation between amylose content and temperature. Umemoto et al. (2008) evaluated a near-isogenic line-NIL(*Wx* ^*a*^) with Kasalath chromosome segment harboring *Wx* ^*a*^ in a Nipponbare genetic background for AC and heading date. NIL(*Wx* ^*a*^) plants flowered 11 days later than Kasalath leading to change in ripening temperature (1.8 ^o^C) and this difference did not contribute to change in amylose content between NIL(*Wx* ^*a*^) (30.7%) and Kasalath (31.5%). In the present study, mean temperatures over 30 days after heading for seven years in the 13 early heading lines ranged from 25.5 °C to 27.6 °C with a mean of 26.3 °C whereas those of 83 lines lacking the *O. rufipogon qDTH6* QTL ranged from 22.9 °C to 25.7 °C with a mean of 24.1 °C. However, difference for heading date, amylose and protein contents between two groups were significant (Table 1) and these results are not consistent with those of previous reports (Umemoto et al. 2002, 2008). And the AC mean value (17.5%) of two lines with the *O. rufipogon* segment harboring the *Wx* ^*a*^ and *qDTH6* genes was not different from that of the other 11 lines (17.5%) with Hwaseong *Wx* ^*b*^ and *O. rufipogon qDTH6* alleles (*P* >0.90) (Additional file 1: Table S1). These results seem to imply that the low AC in the early heading lines with the *Wx* ^*a*^ allele might be due to genotype-by-environment interaction caused by altered heading date. Also, it is possible that additional QTL for AC not detected in this study might be involved in controlling the AC content because a number of studies reported the existence of QTL for AC other than *Wx* locus in this chromosomal region (Edabi et al. 2013; Kwon et al. 2011). To test the genotype-by-environment interaction, we are developing a set of near-isogenic lines with different chromosomal segments around the *Wx* gene.

Most of the QTLs detected in this study were located in the same or adjacent regions as those reported by previous studies, and clustered on several regions (Aluko et al. 2004; Bao et al. 2000, 2004; Kwon et al. 2011; Sabouri et al. 2012; Tan et al. 1999; Zhang et al. 2013). Among three QTLs identified for amylose content, *qAC2* near RM174-RM452 shared a similar genomic location as the one by Tan et al. (1999), and *qAC6* was localized within the QTL mapped by Kwon et al. (2011). *qAC10,* detected in the interval of RM333-RM591 colocalized with *qAC10* detected using the RILs from a cross between ‘Ilpumbyeo’ and ‘Moroberekan’ (Cho et al. 2014). At *qAC10*, the *O. rufipogon* allele decreased amylose content. All six QTLs detected for chalky rice ratio shared the similar locations as the QTLs identified in previous reports (Chen et al. 2011; Li et al. 2003; Tan et al. 2000). Among the six QTLs for head rice ratio, five QTLs were located in the similar locations as the QTL for HR (Aluko et al. 2004; Mei et al. 2002; Nelson et al. 2011; Tan et al. 2001). However, *qHR4* located in the interval between RM401-RM518 appears to be a new QTL. Also, most of the QTLs for the starch viscosity traits shared the similar locations as the QTLs reported in previous studies (Cho et al. 2014; Edabi et al. 2013; Xu et al. 2015; Zhang et al. 2013) except for four QTLs, *qHPV10*, *qBDV12*, *qCPV11* and *qSBV8*. Co-localization of these QTLs, as the result of either pleiotropic effects or close linkage, can provide an explanation for the genetic basis of correlations among the several quality traits tested.

Among five QTLs detected for glossiness of cooked rice, one QTL, *qGCR9,* showed a positive effect on palatability. Glossiness of cooked rice (GCR) is considered as an indirect palatability values based on the significant correlation between sensory test and glossiness of cooked rice (Kwon et al. 2011; Takeuchi et al. 2007). Field trials using introgression lines for 7 years confirmed that ILs containing *O. rufipogon* DNA in the target region between RM242 and RM245 showed significantly higher GCR than NIL with Hwaseong DNA in the same region as well as the parent, Hwaseong. The GCR in the *O. rufipogon* NILs was 11.9–16.4% higher than that of the corresponding Hwaseong NIL, and 8.7–13.1% higher than that of the Hwaseong parent. Higher GCR in the *O. rufipogon* NILs might be associated with protein content based on the finding that the *O. rufipogon* NILs showed lower protein content than the Hwaseong NIL and the parent, Hwaseong (*P* <0.1). Although we failed to declare the existence of QTL for protein content, the protein content QTL detected near *qGCR9* in 2 years 2009 and 2013 exceeded the LOD value of 2.1. Wada et al. (2006) also detected a QTL *qPC9* for protein content near RM1896 on chromosome 9 using recombinant inbred lines from two *japonica* cultivars. Numerous studies reported that protein content was negatively correlated with eating quality (Juliano et al. 1965; Kwon et al. 2011; Wada et al. 2006), as high protein content rice displayed lower adhesiveness and water uptake ratios than low protein content rice (Takeuchi et al. 2007). Also, no significant difference among four lines with/without *O. rufipogon* DNA in the target region between RM242 and RM245 and the parent, Hwaseong was not observed indicating that grain shape traits are not associated with GCR. All these results support the idea that high GCR is associated with low protein content in the *O. rufipogon* ILs in this study. However, the possibility of close linkage of two QTLs for protein content and glossiness of cooked rice in the candidate region of 3.4 Mb cannot be ruled out.

Also, the *O. rufipogon* NILs performed better than the Hwaseong NIL and Hwaseong in spikelets per panicle contributing to an increase grain yield and this result is in good agreement with the previous report (Xie et al. 2008). QTL alleles from wild species that are favorable for some traits may be associated with deleterious effects on other traits (Xiao et al. 1998). In this research, *O. rufipogon* alleles in the target region on chromosome 9 had a favorable effect on yield and GCR, while *O. rufipogon* alleles in this region were independent from undesirable grain traits such as higher amylose content and chalky rice ratio, and low head rice ratio. Additional studies to fine map the region of *qGCR9* and to understand which factors are associated with increased GCR values are underway. Cloning the gene(s) underlying QTL for GCR and grain yield on chromosome 9 is the first step toward understanding how the gene(s) functions and is likely to offer insights into how variation at this region can be manipulated to provide the desired rice quality and yield advantage in *japonica* rice.

The program to transfer useful genes from wild rice into cultivated counterparts is active and aims to increase yield, yield stability, and crop genetic diversity (Brar and Khush 1997; Xie et al. 2008; Suh et al. 2014). However, efforts to improve rice quality of modern cultivars using wild rice as donor parents are limited. In this study, *O. rufipogon* alleles decreased rice quality in the Hwaseong background at most QTLs. However, a few QTLs, including *qGCR9* and *qAC10* for amylose content, were beneficial in the Hwaseong background, revealing the possibility that *O. rufipogon* alleles could improve grain quality traits. No *O. rufipogon* introgression was detected in a few chromosomal regions. For example, 96 ILs did not have any *O. rufipogon* segment on chromosome 5 . It is also possible to identify additional beneficial QTLs in these chromosomal regions. The ILs with beneficial QTL for grain trait could be used as new cultivars and as promising parental lines in rice breeding programs.

## Conclusions

Development of high quality rice cultivars is the top priority in *japonica* breeding programs, therefore quantitative trait loci (QTL) for glossiness of cooked rice and amylose content associated with eating quality have received much research focus because of their importance in rice quality. Our results demonstrate that the *qGCR9* allele from *O. rufipogon* increased glossiness of cooked rice in the *japonica* background while *O. rufipogon* alleles in this region were independent from undesirable grain traits such as higher amylose content and chalky rice ratio, and low head rice ratio. In addition, *O. rufipogon* alleles near the *qGCR9* on chromosome 9 had favorable effects on yield components leading to increase in grain yield. SSR markers linked to *qGCR9* can be used to develop high-quality *japonica* lines and offer a starting point for map-based cloning of genes underlying this trait.

## Methods

### Plant Materials and Field Trials

A set of 96 introgression lines (ILs) were developed from a cross between the Korean elite *O. sativa japonica* cultivar ‘Hwaseong’ and *O. rufipogon* (IRGC 105491) (Cho et al. 2003). Genotypes of the 96 ILs are provided in Additional file 1: Table S1. From 2007 to 2013, 96 ILs and the parents were grown in the experimental fields at Chungcheongnam-do Agricultural Research and Extension Services (CNARES). Germinated seeds were sown in the middle of April, and the seedlings were transplanted in the paddy field about 30 days later. Each line was represented by a single row of 33 plants with 30 × 15 cm spacing in a randomized complete block design with two replicates. Fertilizer (N-P_2_O_5_-K_2_O) was applied at the rate of 90-45-57 kg/ha. After harvest, rice grains were air-dried until the moisture content reached 15% and then dehulled to obtain brown rice. Each brown rice sample was milled to 92% milling yield by a polishing machine (MC-90A, Toyo, Japan), and then milled rice samples were stored in a refrigerator (15 °C) to prevent quality alteration. The polisher was set to stop when 200 g brown rice sample reached 184 g in weight and the 92% milling rate has been used as a standard way for evaluating rice grain quality traits. To analyze amylose, protein content, and starch viscosity characteristics, milled rice samples were ground to fine powder through a 100-mesh screen (Cyclotec, Foss, Denmark).

### Trait Measurements

#### Agronomic Traits

Days to heading (DTH) were evaluated as the number of days from seeding until 50% of the panicles from the 30 plants were heading. The middle 10 plants from each line were chosen for evaluation, and the arithmetic mean of the measurements from each line was used to determine the number of spikelets per panicle (SPP). SPP were measured as the mean of three major panicles per plant. Brown rice yield (GY) was measured as the weight of bulked grain harvested from the 20 plants per line. The brown rice yield was corrected for the 15% grain moisture content. Grain shape traits of four NILs were evaluated to determine their possible association with rice quality traits. Grains with hull were allowed to dry naturally after harvesting, and partial or un-filled seeds were removed with water. Fully filled seeds were re-dried in an oven at 30 °C for 24 h. Thousand-grain weight (TGW) was evaluated by measuring the weight of 50 randomly-selected, fully-filled grains; this method was performed in triplicate and the values were averaged to yield a single mean. Grain length (GL), grain width (GW), grain thickness (GT) and length/width ratio were measured in 200 randomly selected fully filled grains with and without hull using a digimatic caliper (Mitutoyo Corp., Tokyo, Japan). Moisture content of the grain was measured using a Grain Moisture Meter (GMK- 303), and values were again averaged over 3 replications.

#### Rice Appearance Quality Traits

Head rice ratio (HR, unbroken and broken translucent grains with at least 3/4 of a whole grain) and chalky rice ratio (CR, grain with an opaque, chalky appearance covering half or more of the body of the grain) were calculated using an RN-300 (Kett, Japan). Glossiness of cooked rice (GCR) was measured using a sample of 33 g milled rice using a Toyo taste meter (MA-90A and 90B, Toyo, Japan) in accordance with the manufacturer’s instructions. Amylose content was measured using the method by Perez and Juliano (1978), and protein content was analyzed using an automatic Infratec 1241 grain analyzer (Foss, Denmark).

#### Starch Viscosity Characteristics

Starch viscosity characteristics of rice flour were analyzed by using a Rapid Visco-Analyzer (RVA-4, Newport Scientific, Australia). Each rice flour (3.50 ± 0.01 g, based on 12% moisture) was added to 25.0 ± 0.1 g of water (adjusted to correct for sample moisture content) in a new test canister. The paddle was stirred vigorously to disperse the sample. The canister and paddle were inserted into the instrument, and the RVA trace recorded over 12.5 min. Starch viscosity characteristics were described using seven parameters of the pasting curve: Pasting temperature (PST), peak viscosity (PKV), hot paste viscosity (HPV), cool paste viscosity (CPV), breakdown viscosity (BDV = PKV − HPV), setback viscosity (SBV = CPV − PKV), and consistency viscosity (CSV = CPV − HPV). All starch viscosity characteristics were measured in rapid visco units (RVU) with three replications per sample (Yuan et al. 2010).

### DNA Extraction Simple Sequence Repeat (SSR) Analysis and Sequence Analysis

Total genomic DNA was extracted from fresh leaf tissue of each line in bulk according to the chloroform-based DNA extraction protocol (Causse et al. 1994). SSR analysis was performed according to the method described in Panaud et al. (1996). Primer sequences of published rice microsatellite (RM) markers located within the target region were obtained from the Gramene database (http://www.gramene.org, McCouch et al. 2002). For the fine mapping of *qGCR9*, additional SSR markers were identified from the *O. sativa* Nipponbare genome (http://www.gramene.org).

Allelic differences of *Wx* and *ALK* genes on chromosome 6 between ‘Hwaseong’ and *O. rufipogon* were analyzed. For the *Wx* gene of two parents, we used a PCR primer set, WP-A2 and WP-B (Yamanaka et al. 2004). This primer set amplifies the fragment containing the first exon-intron junction of the gene harboring a single-base substitution (AG*G*T to AG*T*T) responsible for different levels of granule-bound starch synthase between two alleles, *Wx* ^a^ and *Wx* ^b^. For the *ALK* gene of two parents, we used a PCR primer set A4F (5′-CATCATACGGGAGAACGACTGGA-3′) and A4R (5′-TCACCATTGGTACTTGGCCTTGA-3′) to generate fragments harboring SNP3 and SNP4 (Gao et al. 2011). PCR was carried out in a 25ul reaction mixture using Takara Emerald Ready Mix and 20 ng of genomic DNA as template. The PCR cycle were: 96 °C for 5 min; 35 cycles of 98 °C for 10 s, 55 °C for 30 s, and 72 °C for 1 min; and final extension at 72 °C for 5 min. The PCR products were electrophoresed on a 1.5% agarose gel (Lonza, USA) and target band was purified from agarose gels and sequenced using the primers A4F and A4R (Solgent, Korea. http://www.solgent.com).

### QTL Mapping and Statistical Analyses

QTL analysis was performed using single point analysis (SPA) and composite interval mapping (CIM) in Q-gene (ver. 4.3.10). CIM analysis was performed with a 10 cM window size. The log-likelihood (LOD) threshold significance level (*P* < 0.05, LOD ≥ 2.1) was determined from 1000 permutations and ranged from 2.1 to 2.4 per trait and year. The existence of QTL was declared if it was detected in more than half the trials over seven (grain quality traits) or 5 years (starch viscosity traits) with the LOD threshold of 2.0. The QTLs reported in this study were commonly detected by both methods (SPA and CIM), and all QTL data from SPA were represented. Multivariate statistical analyses, including *t*-tests, analysis of variance (ANOVA), and correlation analysis of grain quality traits, were performed using SPSS software (ver. 18.0.0). Phenotypic means of each genotype classes were compared using Duncan’s multiple range test. The QTL was named following the nomenclature recommended by McCouch and CGSNL (McCouch and CGSNL 2008).

## Additional file

Additional file 1: Table S1. Genotypes of introgression lines. (XLSX 54 kb)
